# To the Congress

**Published:** 1887-08

**Authors:** 


TO THE CONGRESS.—As an item of interest to those who propose going to Washington to the Medical Congress, we will state that the St. Louis delegates are going via the O. & M. Railroad, and express a desire to have those from the South to join them. Reduced rates have been secured, through trains, and the trip through the beautiful Cheat River country will be made in day time; also, will pass Harper's Ferry in daylight. The excursion will leave St. Louis early Friday morning September 2. Address G. D. Bacon, General Passenger Agent O. & M. R. R., St. Louis.

